# The City of Johannesburg can end AIDS by 2030: modelling the impact of achieving the Fast‐Track targets and what it will take to get there

**DOI:** 10.1002/jia2.25068

**Published:** 2018-01-23

**Authors:** Robyn M Stuart, Nicole Fraser‐Hurt, Cliff C Kerr, Emily Mabusela, Vusi Madi, Fredrika Mkhwanazi, Yogan Pillay, Peter Barron, Batanayi Muzah, Thulani Matsebula, Marelize Gorgens, David P Wilson

**Affiliations:** ^1^ Department of Mathematical Sciences University of Copenhagen Copenhagen Denmark; ^2^ Burnet Institute Melbourne Australia; ^3^ The World Bank Washington DC USA; ^4^ School of Physics University of Sydney Sydney Australia; ^5^ Department of Health Gauteng Province Johannesburg South Africa; ^6^ Department of Health Johannesburg Health District Johannesburg South Africa; ^7^ National Department of Health Pretoria South Africa; ^8^ School of Public Health University of the Witwatersrand Johannesburg South Africa; ^9^ Monash University Melbourne Australia

**Keywords:** Fast‐Track targets, ending AIDS, Johannesburg, HIV modelling, allocative efficiency

## Abstract

**Introduction:**

In 2014, city leaders from around the world endorsed the Paris Declaration on Fast‐Track Cities, pledging to achieve the 2020 and 2030 HIV targets championed by UNAIDS. The City of Johannesburg – one of South Africa's metropolitan municipalities and also a health district – has over 600,000 people living with HIV (PLHIV), more than any other city worldwide. We estimate what it would take in terms of programmatic targets and costs for the City of Johannesburg to meet the Fast‐Track targets, and demonstrate the impact that this would have.

**Methods:**

We applied the Optima HIV epidemic and resource allocation model to demographic, epidemiological and behavioural data on 26 sub‐populations in Johannesburg. We used data on programme costs and coverage to produce baseline projections. We calculated how many people must be diagnosed, put onto treatment and maintained with viral suppression to achieve the 2020 and 2030 targets. We also estimated how treatment needs – and therefore fiscal commitments – could be reduced if the treatment targets are combined with primary HIV prevention interventions (voluntary medical male circumcision (VMMC), an expanded condom programme, and comprehensive packages for female sex workers (FSW) and young females).

**Results:**

If current programmatic coverage were maintained, Johannesburg could expect 303,000 new infections and 96,000 AIDS‐related deaths between 2017 and 2030 and 769,000 PLHIV by 2030. Achieving the Fast‐Track targets would require an additional 135,000 diagnoses and 232,000 people on treatment by 2020 (an increase in around 80% over 2016 treatment numbers), but would avert 176,000 infections and 56,500 deaths by 2030. Assuming stable ART unit costs, this would require ZAR 29 billion (USD 2.15 billion) in cumulative treatment investments over the 14 years to 2030. Plausible scale‐ups of other proven interventions (VMMC, condom distribution and FSW strategies) could yield additional reductions in new infections (between 4 and 15%), and in overall treatment investment needs. Scaling up VMMC in line with national targets is found to be cost‐effective in the medium term.

**Conclusions:**

The scale‐up in testing and treatment programmes over this decade has been rapid, but these efforts must be doubled to reach 2020 targets. Strategic investments in proven interventions will help Johannesburg achieve the treatment targets and be on track to end AIDS by 2030.

## 1 Introduction

In 2014, city leaders from around the world endorsed the Paris Declaration on Fast‐Track Cities, pledging to achieve the 2020 and 2030 HIV targets championed by UNAIDS 1. The Fast‐Track targets, now ubiquitous in the HIV field, state that by 2020, 90% of all people living with HIV (PLHIV) will know their HIV status, 90% of all people with diagnosed HIV infection will receive antiretroviral therapy (ART), and 90% of all people receiving ART will be virally suppressed (90‐90‐90 targets), with these percentages increasing to 95% by 2030 (95‐95‐95 targets). The 90‐90‐90 and 95‐95‐95 targets are associated with epidemiological milestones: approximately 80% to 90% reductions in new infections and AIDS‐related deaths by 2030, often considered synonymous with the goal of ending AIDS 2.

**Figure 1:**
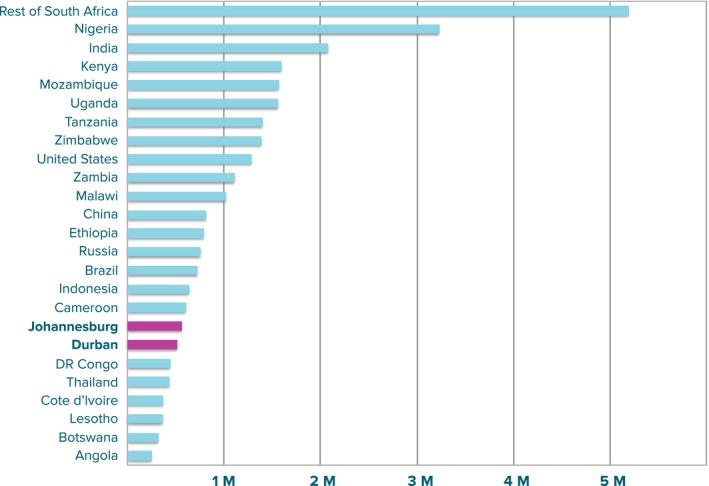
Estimated number of PLHIV in 2013 (UNAIDS 5). Johannesburg and Durban have been disaggregated from the rest of South Africa and are highlighted in pink. Together, the two cities make up 3% of the estimated global burden of HIV, with Johannesburg alone accounting for around 9% of South Africa's HIV burden.

The City of Johannesburg became a signatory to the Paris declaration in March 2016 along with 19 other municipalities in South Africa, joining other cities around the world 3. The crucial role that cities will play in achieving the Fast‐Track targets 4 is particularly relevant in South Africa; both Johannesburg and Durban metropolitan municipalities are estimated to have more than 500,000 PLHIV, which would qualify them for positions in the top 25 countries in the world according to HIV burden if they were counted alongside nations (2013 UNAIDS estimates 5; Figure 1). In this study, our focus is on the City of Johannesburg.

Notwithstanding the considerable political will that Johannesburg's leaders demonstrated in signing the Paris Declaration, it remains uncertain how to translate the Fast‐Track targets into programmatic or financial stratagems. The translation step calls for an analysis of the HIV care and treatment cascade. Over the past 5 years, the HIV care and treatment cascade has been established as a useful framework for assessing the gaps in accessing the full range of diagnostic, care and treatment services available for PLHIV 6, 7, 8. Understanding and minimizing blockages and leakages along the cascade is the only way that the Fast‐Track targets can be attained 9, 10.

The HIV response within South Africa as a whole is heavily focused on the achievement of the Fast‐Track targets, with many service delivery modalities in place to improve diagnosis, linkage to and retention in care, and treatment initiation, monitoring and adherence. Despite the recognition of the importance of cities in achieving the Fast‐Track targets, most studies tend to focus on analysing the care and treatment cascade within countries 7, 9, 11, 12, 13 or within clinics^14^, ^15^. Aside from the strategic importance of cities, an analysis of a city's cascade can shed light on how well the network of clinics is working together as a whole. Mathematical models have proven useful in answering questions of this type, and a multitude of different modelling frameworks have been developed in response, both specifically for South Africa 16 and more generally 17.

To understand what it will take for the City of Johannesburg to achieve the Fast‐Track targets, we adapted the Optima HIV epidemic and resource allocation model in order to capture the key aspects of the HIV care and treatment cascade 18. Although the Optima HIV model has been successfully applied in many countries to assess the impact and optimize the allocation of HIV programme spending 19, it has yet to be used for a detailed analysis of the care and treatment cascade, largely due to a lack of comprehensive data. For this analysis, we sought data from a number of sources, including clinic‐level data, cohort studies, national reports, and a novel record‐linkage analysis providing comprehensive viral load and CD4 data for Johannesburg. Using these data in our specially tailored model, we estimate what it would take in terms of programmatic targets and investments for the City of Johannesburg to meet the Fast‐Track targets, and then estimate the epidemiological impact that this would have.

## 2 Methods

### 2.1 Model structure

**Figure 2:**
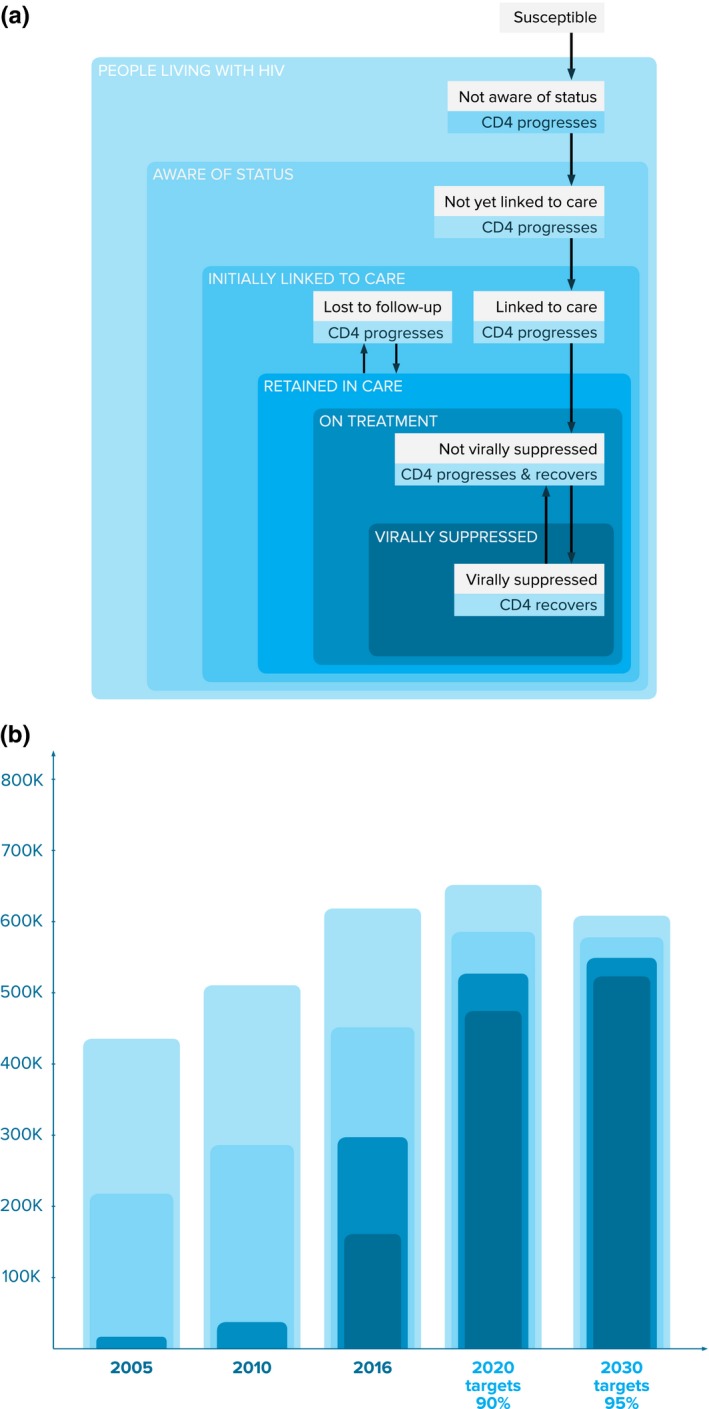
(a) The compartmental structure of the Optima HIV epidemic model, adapted to account for the care and treatment cascade; (b) Progress towards the Fast‐Track targets over 2005–2016 (first 3 columns), and model estimates of the scale‐up required to 2020 and 2030 (final 2 columns).

We adapted the deterministic compartmental epidemic model structure of Optima HIV 18, making several modifications in order to better capture aspects of the care and treatment cascade (Figure 2a). Each population group included in the model has 37 possible health states: 7 treatment‐related states (susceptible, undiagnosed, diagnosed, in care, receiving ART and not virally suppressed, receiving ART and virally suppressed, lost‐to‐follow‐up), with all infected stages further disaggregated into 6 CD4‐related states (acute HIV infection, >500 cells/μL, 350 to 500 cells/μL, 200 to 350 cells/μL, 50 to 200 cells/μL, <50 cells/μL). In addition, we disaggregated the total population of the City of Johannesburg (estimated at 4.9 million in 2016) into 26 sub‐populations: males and females aged 15 to 60 stratified into 5‐year age bands, plus infants aged 0 to 2, children aged 3 to 14, males and females aged 60+, female sex workers (FSW), clients of female sex workers, men who have sex with men, and people who inject drugs.

### 2.2 Model transitions

The movement of people along the cascade is modelled by the black arrows depicted in Figure 2a. Each arrow is associated with a probability of transitioning to a different stage of the care continuum within a fixed interval of time, and each is specific to a particular sub‐population and CD4‐related state. Following the surveillance study undertaken by Howard University in conjunction with the President's Emergency Plan For AIDS Relief (PEPFAR) and the National Department of Health, we consider someone to be linked to care if they have been enrolled in a facility for continuum of HIV care and have completed and received the results of an initial ART eligibility assessment within 3 months of diagnosis 20. The different transition probabilities associated with different CD4 counts are primarily intended to distinguish the behaviour of people with <200 CD4 cells/μL (AIDS‐stage infection) and also capture differences in mortality rates. We assume that people with AIDS‐stage infection have a symptomatic testing rate above the standard testing rate for their population cohort, which gives us an estimated median CD4 count at diagnosis of 340 cells/μL in 2015, approximately on par with other studies 21, 22. We assume that treatment spots are first taken by those with lower CD4 counts, which give us a mean CD4 count at time of treatment initiation of 190 cells/μl, again comparable to other studies 21. In addition to the transitions along the cascade, we also take into account transitions between different sub‐populations (for example, mixing between adult female and FSW populations) and disease states (as stratified by CD4 counts).

### 2.3 Data

The data used to inform the model's transition probabilities are summarized in Table 1. Details on the model equations are provided in the Supplementary Material, and full details of the model parameters are provided in the Optima HIV parameters sources compendium 23. Estimating the current state of the care and treatment cascade relied heavily on the Howard University surveillance study 20.

**Table 1:** Key parameters used to inform the transitions for the epidemiological model

Transitions	Data types	Data availability	Value
Cascade			
Infection	Sexual behavioural data (number of acts per year & probability of condom use with regular, casual and commercial partners)	National	Time‐varying & population‐specific
	Injecting behavioural data (number of injections per year & probability of syringe sharing)	National	Time‐varying & population‐specific
	Intervention uptake (% of people accessing PrEP, circumcision, ART, OST & PMTCT)	Municipal	Time‐varying & population‐specific
	Per‐act transmission probabilities	Literature	See Supplementary Materials
	Efficacy of interventions	Literature	See Supplementary Materials
	Partnership formation patterns	National	Time‐varying & population‐specific
Diagnosis	% of population tested for HIV in the last 12 months	National	Time‐varying & population‐specific
Linkage to care	% of people linked to care within 3 months of diagnosis	Municipal	Time‐varying & population‐specific
Treatment initiation	Matched to available data on the number of people on ART	Municipal	Time‐varying & population‐specific
Viral suppression	Average time taken from treatment initiation to viral suppression; frequency of viral load monitoring	Municipal	Time‐varying
Treatment failure	% of those who were virally suppressed at their last VL test	Municipal	Time‐varying
Loss to follow‐up	% of people not returning to their clinic after 90 days	Clinic	Time‐varying
CD4 change			
CD4 progression	Duration of acute infection	23	0.24 [0.10, 0.30] years
	Time to move from CD4 > 500 to 350 < CD4 < 500	23	0.95 [0.62, 1.16] years
	Time to move from 350 < CD4 < 500 to 200 < CD4 < 350	23	3.00 [2.83, 3.16] years
	Time to move from 200 < CD4 < 350 to 50 < CD4 < 200	23	3.74 [3.48, 4.00] years
	Time to move from 50 < CD4 < 200 to CD4 < 50	23	1.50 [1.13, 2.25] years
CD4 recovery on suppressive ART	Time to move from 350 < CD4 < 500 to CD4 > 500	23	2.20 [1.07, 7.28] years
	Time to move from 200 < CD4 < 350 to 350 < CD4 < 500	23	1.42 [0.90, 3.42] years
	Time to move from 50 < CD4 < 200 to 200 < CD4 < 350	23	2.14 [1.39, 3.58] years
	Time to move from CD4 < 50 to 50 < CD4 < 200	23	0.66 [0.51, 0.94] years
	Time from treatment initiation to achieve viral suppression	23	0.20 [0.10, 0.30] years
CD4 progression & recovery on non‐suppressive ART	% moving from CD4 > 500 to 350 < CD4 < 500 per year	23	2.60 [0.50, 27.50]%
	% moving from 350 < CD4 < 500 to CD4 > 500 per year	23	15.00 [3.80, 88.50]%
	% moving from 350 < CD4 < 500 to 200 < CD4 < 350 per year	23	10.00 [2.20, 87.00]%
	% moving from 200 < CD4 < 350 to 350 < CD4 < 500 per year	23	5.30 [0.80, 82.70]%
	% moving from 200 < CD4 < 350 to 50 < CD4 < 200 per year	23	16.20 [5.00, 86.90]%
	% moving from 50 < CD4 < 200 to 200 < CD4 < 350 per year	23	11.70 [3.20, 68.60]%
	% moving from 50 < CD4 < 200 to CD4 < 50 per year	23	9.00 [1.90, 72.30]%
	% moving from CD4 < 50 to 50 < CD4 < 200 per year	23	11.10 [4.70, 56.30]%
Population transitions			
Risk	Average length of time spent as sex worker	National	12 years
	Average length of time spent as client of sex worker	National	15 years
Age	Defined by width of age bins	N/A	

The transitions between CD4 categories were determined following extensive literature review and data synthesis; full details are contained in the Supplementary Materials. The transitions between cascade stages were informed by local data.

### 2.4 Model calibration

We initialized the model in 2000 and produced projections from 2000 to 2030. We fitted the model to data on population sizes, HIV prevalence and the number of people on ART by adjusting a subset of the model's parameters in order to minimize the mean absolute percentage error between the model's estimates and the data, and then subjecting the projections produced by the model to scrutiny and validation by the district, province and national health departments involved in the study. For the purposes of fitting, we specified which of the model's parameters could be adjusted, as it is neither feasible nor desirable to allow all parameters to vary freely. Since our focus is on modelling the care and treatment cascade, we selected parameters to match the cascade transitions depicted in Figure 1. Specifically, we fitted: 1 the initial HIV prevalence in each sub‐population; 2 parameters controlling the probability of infection; 3 testing rates (within 10% of reported values); 4 the percentage of people linked to care within 3 months (within 10% of reported values); 5 the average time taken from treatment initiation to viral suppression, within the ranges given in Table 1; 6 the proportion of people virally suppressed at their last test (within 10% of reported values); 7 the proportion of people not returning to their clinic after 90 days and 8 all CD4 progression and recovery rates, within the ranges given in Table 1. The key outputs of the calibration are included in the Supplementary Material. The model's estimates of prevalence, infections, deaths, number of people on ART and PLHIV are generally aligned to available data.

### 2.5 Model analysis

We estimated the HIV testing and treatment scale‐up required to meet the Fast‐Track targets under a range of assumptions about coverage levels of other programmes. As the baseline scenario, we assume that coverage of all other programmes would remain at latest reported levels. The testing and treatment scale‐up required under this baseline scenario is shown in Figure 2b. We then constructed alternative scenarios to describe plausible programmatic scale‐ups, informed by South Africa's National Strategic Plan (NSP) for 2017 to 2022 24 and the South African National Sex Worker HIV Plan for 2016 to 2019 25.

In modelling the impact of the 2017 to 2022 NSP, we restrict our attention to Goal 1 (of a total 8 goals), which aims to reduce new HIV infections through combination prevention interventions, and more specifically to sub‐objective 1.1.2, the component of Goal 1 that outlines an approach for the provision of targeted biomedical HIV prevention services. (Sub‐objectives 1.1.1 and 1.1.3 relate to education programming, which is outside of the scope of our modelling framework; sub‐objective 1.1.4 relates to the provision of PrEP, which we model separately as part of the National Sex Worker Plan; and sub‐objective 1.1.5 relates to PMTCT, which we include in the treatment scale‐ups associated with the Fast‐Track targets.) We omit Goal 2 because it relates to the attainment of the Fast‐Track targets (which we already model separately). We omit Goal 3, which aims to reach all key and vulnerable populations with customized and targeted interventions, because concrete targets are yet to be formulated for many of the indicators in the NSP's Monitoring and Evaluation Framework. Finally, we omit Goals 4–8 because these relate to addressing the social and structural drivers of the epidemics, grounding the responses in a human rights approach, promoting leadership, mobilizing resources and strengthening strategic information, and while these are essential components of the strategy, our modelling framework is best suited to analysing the impact of biomedical and behavioural interventions designed to directly impact on one of the proximal determinants of HIV transmission or mortality.

For each scenario, we calculated the coverage and estimated investment levels required to attain the Fast‐Track targets, as well as the impact.

## 3 Results

### 3.1 Progress towards Fast‐Track targets

We estimate that in 2016 there were 616,000 PLHIV in Johannesburg, with 73% aware of their status, 77% of those diagnosed linked to care (with 57% of newly diagnosed people linked to care within 3 months), 80% of those who were initially linked to care retained in care (84% of whom are receiving treatment) and 54% of those receiving treatment with viral suppression. Using the UNAIDS cascade with PLHIV as denominator (which translates the Fast‐Track targets to 90%‐81%‐72%), the achievement of the three 90s in 2016 was 73%‐48%‐26%. The care and treatment cascade improved significantly over the past 11 years across all stages for which we have data (Figure 2b). The proportion of people aware of their status was estimated to increase from 50% to 73%, attributable to significant increases in testing programme coverage throughout the entire country 26. In particular, the proportion of MSM aware of their status was estimated to increase from 30% to 63%, and in FSW the increase was even more marked (from 30% to 70%, consistent with other estimates 25, 27). Expanded treatment eligibility and major investments in treatment increased the proportion of diagnosed people receiving treatment from 8% to 64% (although treatment coverage remained low among FSW, at 23% 26, 27).

### 3.2 What will it take to achieve the Fast‐Track targets?

We estimate that achieving the Fast‐Track targets would require an additional 135,000 HIV diagnoses and 232,000 people on treatment by 2020 relative to 2016 (an increase in around 80% over 2016 treatment numbers; see Figure 2b). Using unit cost estimates from the South African HIV and TB Investment Case 28 in combination with the model's estimates of the programme coverage targets required to reach the first two stages of the 90‐90‐90 and 95‐95‐95 targets, we broadly estimated total investment requirements of ZAR 6.94 billion (USD 0.51 billion) over the 4 years to 2020, and ZAR 32.1 billion (USD 2.37 billion) over the 14 years to 2030 (Tables 2 and 3). Taking into account the proportion of the population living in Johannesburg, our estimates of Johannesburg's investment requirements to 2020 are commensurate with those provided in the 2017 to 2022 NSP^24^. Around 10% of total investments would be required for testing programmes and the remainder for funding significant expansions to treatment and care programmes.

**Table 2:** Coverage and investment levels required to achieve the first two 90 targets and the first two 95 targets under different assumptions about prevention programme coverage

	2016	2017	2018	2019	2020	Annual average 2021–2030	Total 2017–2030
Achieving 90% aware by 2020 and 95% aware by 2030							
Adult testing rates	51%	58%	65%	72%	80%	80%	–
Target population (millions)	3.8	3.9	4.0	4.1	4.2	4.3	59.2
Number of tests required (millions)	1.9	2.3	2.6	3.0	3.4	3.4	45.3
Investment required (ZAR millions)	183	213	245	278	317	183	2,883
Requirements to achieve 90% on treatment by 2020 and 95% by 2030 without scale‐up in other prevention programmes							
Current prevention programme coverage maintained							
Number required on ART (millions)	0.299	0.368	0.419	0.474	0.530	0.568	7.474
Investment required (ZAR millions)	1,161	1,426	1,626	1,838	2,055	2,205	28,999
Requirements to achieve 90% on treatment by 2020 and 95% by 2030 with VMMC scale‐up							
Number required on ART (millions)	0.299	0.366	0.416	0.468	0.521	0.556	7.331
ART investments required (ZAR millions)	1,160	1,422	1,612	1,814	2,020	2,158	28,445
Requirements to achieve 90% on treatment by 2020 and 95% by 2030 with VMMC and condom distribution scale‐up							
Number required on ART (millions)	0.299	0.367	0.416	0.468	0.521	0.549	7.265
ART investments required (ZAR millions)	1,160	1,422	1,614	1,816	2,020	2,132	28,188
Requirements to achieve 90% on treatment by 2020 and 95% by 2030 with VMMC and condom distribution scale‐up + FSW strategy							
Number required on ART (millions)	0.299	0.367	0.416	0.468	0.519	0.542	7.189
ART investments required (ZAR millions)	1,160	1,422	1,614	1,814	2,016	2,103	27,893

**Table 3:** Summary of the total investments required to achieve the first two 90 targets and the first two 95 targets, as well as the savings made under different assumptions about prevention programme scale‐up and the impact in terms of infections averted

	Current prevention programme coverage maintained (base)	VMMC scale‐up	VMMC and condom distribution scale‐up	VMMC and condom distribution scale‐up + FSW strategy
Investment in ART & HTC to achieve 90/90 & 95/95, 2017–30 (ZAR m)	31,882	31,328	31,071	30,776
Cost of prevention programme scale‐up, 2017–30 (ZAR m)	–	266	1,321	Not estimated
Savings in ART & HTC programmes relative to base, 2017–30 (ZAR m)	–	554	811	1,106
Net savings relative to base, 2017–30 (ZAR m)	–	288	−510	Not estimated
Infections averted relative to base, 2017–30	–	4%	8%	14%

### 3.3 The impact of achieving the Fast‐Track targets

**Figure 3:**
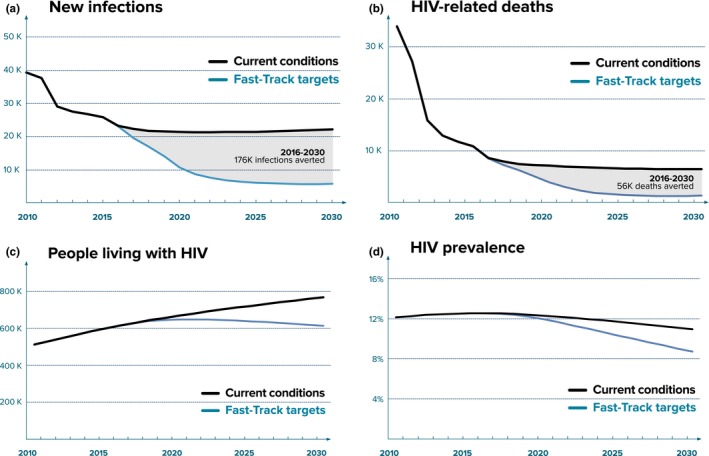
Key epidemic indicators assuming current programme coverage maintained and Fast‐Track targets are met by scaling up HIV diagnosis, treatment and viral suppression, with all other programmes maintained at their latest reported coverage levels.

If the status quo were maintained across the care and treatment cascade (i.e. if current programmatic coverage were maintained such that the proportions of those diagnosed, in care, receiving treatment and virally suppressed remained constant), we estimate that Johannesburg could expect 303,000 new infections and 96,000 AIDS‐related deaths between 2016 and 2030, such that there would be around 769,000 PLHIV by 2030 (Figure 3; base case). In contrast, achieving the 2020 and 2030 Fast‐Track targets would avert 177,000 infections and 56,500 deaths by 2030 (reductions of around 58%), leading to a 26% reduction in the number of PLHIV in 2030 compared to baseline and a 2.2%point reduction in prevalence compared to baseline (Figure 3). While the treatment scale‐up that would be required in order to meet the 90‐90‐90 targets is significant, it is on par with the treatment scale‐up that took place over the first half of the decade (Figure 2b). Furthermore, achieving the 90‐90‐90 targets by 2020 would mean that 95‐95‐95 targets were within reach.

### 3.4 Scaling up primary prevention programmes reduces the cost of reaching the Fast‐Track targets: impact of biomedical prevention service scale‐up

We considered the impact of implementing the targeted biomedical prevention services outlined in sub‐objective 1.1.2 of South Africa's National Strategic Plan (NSP) for 2017 to 2022. This sub‐objective outlines a comprehensive approach for the implementation of targeted biomedical prevention services, with 5 components related to the provision of HIV testing and counselling (HTC), 2 related to the voluntary medical male circumcision (VMMC) programme, and a final component promising the provision of male and female condoms (plus compatible lubricant) in all public and private health facilities, in secondary schools, tertiary institutions, non‐traditional community settings^24^. Since the Fast‐Track targets subsume the HTC targets, we do not model this separately here. Rather, we model the impact of scaling up the VMMC programme and the male and female condom distribution programmes in line with national targets, which aim for 2.5 million medical circumcisions over 2017 to 2020 and the annual distribution of 850 million male condoms and 40 million female condoms. Assuming that the national targets are proportional to population size, we translate these to targets for Johannesburg of 220,000 medical circumcisions over 2017–2020, and annual provision of 68 million male condoms and 3.2 million female condoms. On programme efficacy, we assume that circumcision reduces the probability of HIV‐negative males acquiring infection by 58% 23 and that reaching the condom distribution targets would result in condoms being used in 80% of acts between casual partners (compared to 50% in 2012; see Table S1 and Figure S2 in Supplementary Material for calculations).

We calculate that reaching the VMMC target would require an investment of ZAR 266 million (USD 19.7 million) over 4 years, while reaching the condom distribution targets would require annual investment of ZAR 94 million (USD 6.9 million) (see Table S1 in Supplementary Material for calculations). In combination, attaining these two targets by 2020 and thereafter maintaining them would reduce cumulative new infections between 2017 and 2030 by a further 9% compared to a base case scenario in which coverage of these two programmes is maintained at current levels. This would imply ZAR 811 million (USD 59.9 million) in savings to the cumulative treatment budget to 2030 (Table 3). Investing in the VMMC programme in particular is cost saving: we find that scaling up VMMC alone would deliver a ZAR 554 million (USD 40.1 million) saving to the overall treatment budget out to 2030, and thus would deliver modest net savings even after accounting for the cost of scale‐up.

### 3.5 Impact of implementing the National Sex Worker HIV Plan

We consider the impact of the South African National Sex Worker HIV Plan 2016 to 2019 25. In light of the significant body of evidence demonstrating the impact of structural interventions on the health service use and uptake of biomedical services among sex workers^29^, ^30^, we would like to capture the impact of the structural elements of the package, notably the Human Rights Package (comprising law reform, decriminalization, legal literacy and legal service use), the Social Capital Building Package (comprising community empowerment, and collectivization), and the Economic Empowerment Package (comprising skill building, career‐path defining, and participation in cooperatives and education interventions). Full‐scale financing and implementation of these structural packages in combination with the implementation of the Health Care package is associated with outcomes of: 1 95% condom use in FSW/client commercial acts in 2020, and 2 provision of PrEP to 3000 HIV‐negative FSWs in 2016, with coverage then extended to all HIV‐negative FSWs. Assuming that the national targets are proportional to population size, we translate the latter target to mean that 240 HIV‐negative FSWs in Johannesburg would be provided with PrEP in 2016 (4% coverage), scaling up to 4800 by 2020 (80% coverage). If implemented in addition to the scale‐up of HIV diagnosis and treatment programmes needed to attain the Fast‐Track targets and the planned scale‐up to VMMC and condom distribution services, we estimate that implementing the SW strategy would reduce cumulative new infections between 2016 and 2030 by a further 7%, and would mean that the Fast‐Track targets would be attainable with ZAR 295 million (USD 21.9 million) less required for total treatment investment (Table 3). Unfortunately we are unable to determine cost estimates for the structural elements of the package due to a lack of cost and effectiveness data, so we are unable to determine the magnitude of savings overall.

## 4 Discussion

The results presented in this paper highlight the tremendous impact that achieving the Fast‐Track targets would have on the HIV epidemic in the City of Johannesburg, while also demonstrating the still‐crucial role that primary prevention programmes play in both reducing HIV transmission and in reducing the future financial burden of achieving the Fast‐Track targets. We focused on estimating the investments that would be required in order to get 90% of people diagnosed by 2020 and 90% of those diagnosed onto treatment (with those percentages increasing to 95% by 2030) under different assumptions around prevention programme scale‐up. We also identified key leakages along the cascade that currently present barriers to achieving the Fast‐Track targets, with key problem areas being the rate of linkage to care within 3 months and the rate of viral suppression among those receiving treatment. The state of the care and treatment cascade in Johannesburg has improved greatly over first half of this decade, but there is still significant work to be done if the Fast‐Track targets are to be achieved, and these areas should be particular focal points.

Johannesburg's Implementation Plan for 2016/17 is a testament to the health authorities' commitment to addressing the programmatic scale‐up implied by the Fast‐Track targets. HTC will be promoted in many different ways: through campaigns in high‐transmission areas and among key populations, by making HTC access more convenient (e.g. alternative opening times of services, mobile provision) and integrated (e.g. reinvigorated provider‐initiated counselling and testing, HTC in clinic waiting areas, improved index testing), through approaches to diagnose more men (e.g. targeting factories, transport hubs, tertiary institutions) and by focusing on yield through continuous monitoring and adjustment of efforts. Counsellors will be distributed according to need and clinics' HTC targets, and the electronic ART patient record system will be expanded to include HTC data. Equally important is the authorities' scale‐up efforts to link diagnosed HIV cases to care and treatment (which will help with the estimated need of putting an additional 232,000 people on treatment by 2020). Given that linkage to care is especially challenging if HTC is provided at community level, various providers are tasked with facilitating this process, using the Department of Health's ward based teams and civil society organizations. Adequate supply of HIV drugs and prevention of stock‐outs using the new “Stock Visibility Solution” infrastructure are also in the plan. In order to support adherence, differentiated care options are being or have been introduced, including decentralized medicine delivery schemes, adherence clubs, fast queuing, tracing of those lost to care, and enhanced adherence counselling. This will be supported by improved data systems to identify patients in need of laboratory monitoring, drug refill and additional adherence support. In parallel, scale‐up plans are in place for other core HIV prevention services, including the National Sex Worker HIV plan, plans for enhanced condom distribution based on evidence of need and stock management, and increasing involvement of general practitioners to provide free VMMC services.

We note several limitations to the analyses presented. First, limitations in data availability and reliability can lead to uncertainty surrounding projected results, and these uncertainties were not quantified. Second, many of the Optima HIV model parameters (most notably those related to transmission probabilities, disease progression and programme efficacy) were sourced from clinical and research studies, and may differ from the values that would be observed in Johannesburg. Third, our estimates of the impact of biomedical prevention service scale‐up assume that the planned investments in condom and VMMC scale‐up would be met with increased condom use and increase rates of medical circumcision. Particularly with respect to condom usage, this may be an optimistic assumption as behaviour changes of this type can be difficult to attain in practice. Fourth, our analysis of the costs assumes that all programmes continue to operate at current levels of efficiency, and does not consider the effect of potential efficiency gains. There are various studies that have highlighted the potential for efficiency gains in the implementation of prevention programmes 31, including specific studies on HTC 32, VMMC 33, condom distribution programme 34, and sex worker programme 35 efficiency in various contexts in sub‐Saharan Africa. More functional integration of HIV services, as is currently happening in South Africa within the Integrated Chronic Disease Management and Chronic Treatment Adherence guidelines, creates economies of scope and may also improve the client experience. Technical efficiency gains, in areas where large volumes of patients are to be served such as Johannesburg, are feasible to gain economies of scale and reduce unit costs (e.g. South Africa's task shifting to lower level health cadres, price reductions of drugs and diagnostic tests). Also, efficiency gains may be made through better targeting of programmes, the use of different service delivery modalities, or cutting back on indirect programme costs. It is therefore worth investigating whether such efficiency gains may be made here. However, while there may be further cost‐savings possible in the Johannesburg HIV programme in the future, some are likely offset by the effort required to identify additional and harder‐to‐reach cases and get them linked to HIV care and virally suppressed. HIV testing yield illustrates this challenge of case identification well: in South Africa, six people needed to be tested in 2005 to find one new HIV case in 2005; in 2015 this increased to testing 18 people 36. Another factor potentially offsetting the savings gained through efficiencies is the need to switch more ART clients to second line HIV treatment, which remains more costly.

The importance to Johannesburg of meeting the Fast‐Track targets–thereby averting an estimated 177,000 infections and 56,500 deaths by 2030 and reducing the number of people living with HIV by a third – cannot be overemphasized. Ensuring scale and quality of the HIV treatment programme is vital for the city's economic prosperity and for South Africa as a whole. Johannesburg's commitment towards the Fast‐Track targets echoes the national dedication to achieving the targets and ending AIDS by 2030. “Universal test and treat” (UTT) became national policy in September 2016, with accelerated efforts being made for HIV clinic decongestion, down‐referral of HIV clients, decentralization of services and community‐based monitoring to facilitate UTT. Substantial investments continue to be made throughout South Africa to support HIV testing, linkage to care, pre‐ART care, and treatment initiation, maintenance and adherence. The focus on diagnosing HIV‐positive individuals over recent years has paid off with large increases in the proportion of people aware of their status, and South Africa has also been successful in initiating large numbers of people onto ART. However, significant work remains to be done on a national scale, particularly with respect to improving retention and adherence rates.

Enhanced analysis of the HIV care cascade is crucial for guiding the large investments that will be required to achieve the Fast‐Track targets. In particular, translating broad political targets into actionable stratagems requires detailed knowledge of the current status of the care and treatment cascade, as well as an understanding of the likely future trajectories of the HIV epidemic. There is scope for mathematical models to help with the latter, and for improved data collection and analysis to help with the former. Conducting analyses at a city level means that goals can be mapped against operational budgets, and translated into targets for the clinical and service provision networks. Such analyses would be best conducted within a broader national context, in order to ensure coherence across different jurisdictional levels. Our modelling methodology would be well‐suited for many other cities, especially those who have committed to achieving the Fast‐Track targets, and could be used to answer similar questions about resource needs and impact in a range of settings.

## 5 Conclusions

Enhanced analysis of the HIV care cascade is crucial for guiding the large investments that will be required in order to achieve the goal of ending AIDS by 2030. By providing annual denominators of people living with HIV and requiring services of HIV testing, linkage, treatment initiation and treatment maintenance, this analysis hopes to inform target setting for the Fast‐Track response and as a baseline for UTT. For Johannesburg, getting HIV service delivery right – guided by ruthless data tracking and enhanced analysis – in an era of prolonged and increasing urbanization and rapidly growing ART programme costs is vital both for the individuals who live there, and for the economy as a whole.

## Competing interests

The authors declare no competing interests.

## Authors' contributions

RMS and NF‐H wrote the manuscript. Analyses were carried out by RMS and NF‐H, with substantial technical inputs and data provided by CCK, EM, VM, FM, BM and TM. Supervision, oversight and guidance was provided by YP, PB, MG and DPW.

## Funding

Funding for this study was provided by the World Bank Group and National Health and Medical Research Council.

## Supporting information


**Figure S1**. shows the model estimates of HIV prevalence in each sub‐population compared to available data.
**Table S1.** Calculations to determine the cost of scaling up the VMMC and condom distribution programmes in Johannesburg
