# Supplementary material for: The City of Johannesburg can end AIDS by 2030: modelling the impact of achieving the Fast‐Track targets and what it will take to get there
Source: J Int AIDS Soc. 2018 Jan 23;21(1):e25068. doi: 10.1002/jia2.25068 (PMC5810342; doi:10.1002/jia2.25068)
Supplement: Supplementary file 1 — Figure S1. shows the model estimates of HIV prevalence in each sub‐population compared to available data. Table S1. Calculations to determine the cost of scaling up the VMMC and condom distribution programmes in Johannesburg [file JIA2-21-e25068-s001.docx]

Supplementary material

**Table of Contents**

1. Equations translating data to transition probabilities 1

1.1. CD4 transitions 1

1.1.1. People not on treatment 1

1.1.2. People on suppressive ART 2

1.1.3. People on unsuppressive ART 2

1.2. Cascade transitions 2

1.2.1. Infection 2

1.2.2. Diagnosis 2

1.2.3. Linkage to care 2

1.2.4. Treatment initiation 2

1.2.5. Viral suppression 2

1.2.6. Treatment failure 2

1.2.7. Loss to follow-up 2

1.3. Population transitions 2

1.3.1. Risk transitions 2

1.3.2. Age transitions 2

2. Calibration output 3

3. Intervention cost calculations for VMMC & condoms 4

4. References 5

**List of Figures**

Figure S1: Model estimates of HIV prevalence in each population 20

Figure S2. Assumed relationship between condom distribution and condom use at last sex with casual partner 21

**List of Tables**

# 1. Equations translating data to transition probabilities

## 1.1. CD4 transitions

### 1.1.1. People not on treatment

The time required for people to transition from a compartment with a higher CD4 count to a compartment with a lower CD4 count is assumed to be exponentially distributed with mean times given in Section 1.3 of the Optima HIV parameter sources compendium [[1](#_ENREF_1)].

### 1.1.2. People on suppressive ART

The time required for people to transition from a compartment with a lower CD4 count to a compartment with a higher CD4 count is assumed to be exponentially distributed with mean times given in Section 1.4 of the Optima HIV parameter sources compendium [[1](#_ENREF_1)].

### 1.1.3. People on unsuppressive ART

The probabilities of transitioning from any given compartment to a compartment with either a higher or a lower CD4 count are given in Section 1.5 of the Optima HIV parameter sources compendium [[1](#_ENREF_1)].

## 1.2. Cascade transitions

### 1.2.1. Infection

Details of how the probability of infection is calculated are given in Kerr et al [[2](#_ENREF_2)].

### 1.2.2. Diagnosis

Details of how the probability of diagnosis is calculated are given in Kerr et al [[2](#_ENREF_2)].

### 1.2.3. Linkage to care

We collect data on the average time after diagnosis before people are linked into care. We then assume that the time required for people to be linked to care is exponentially distributed with this mean.

### 1.2.4. Treatment initiation

The probability of treatment initiation is determined by the number of treatment spots available. These spots are first filled by those with lower CD4 counts.

### 1.2.5. Viral suppression

The time required for people to achieve viral suppression after initiating treatment is assumed to be exponentially distributed with mean times given in Section 1.6 of the Optima HIV parameter sources compendium [[1](#_ENREF_1)].

### 1.2.6. Treatment failure

The probability of treatment failure is given in Section 1.6 of the Optima HIV parameter sources compendium [[1](#_ENREF_1)].

### 1.2.7. Loss to follow-up

We collect data on the proportion of people who do not return to their clinic after 90 days, and adjust for the number who died or were known to move to another clinic, to determined the rate of loss to follow-up.

## 1.3. Population transitions

### 1.3.1. Risk transitions

We collect data on the average length of time that people spend in sex work and as clients. We then assume that the time that people spend in these populations is exponentially distributed with this mean.

### 1.3.2. Age transitions

Age transition rates are determined by definition of the width of the age bins.

# 2. Calibration output

Figure S1 shows the model estimates of HIV prevalence in each sub-population compared to available data.


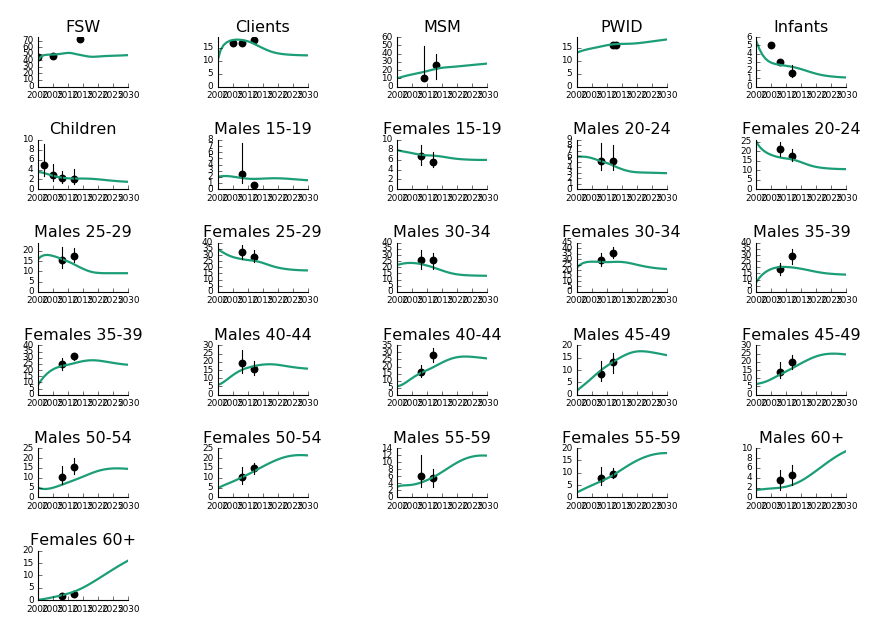


Figure S1: Model estimates of HIV prevalence in each population

# 3. Intervention cost calculations for VMMC & condoms

Calculations to determine the cost of scaling up the VMMC and condom distribution programs are summarized in Table S1.

|  | Intervention specifics | Value | Notes/Source |
| --- | --- | --- | --- |
|  | **VMMC** |  |  |
| A | MMCs required to attain target coverage (2017-2020) | 220,000 | [[3](#_ENREF_3)] |
| B | Cost per MMC | 1,210.19 | [[4](#_ENREF_4)] |
| C | Required investment to attain VMMC targets | **266,241,800** | Calculation: A*B |
|  |  |  |  |
|  | **Condom distribution** |  |  |
| D | Targeted national male condom distribution, 2020 | 850,000,000 | [[5](#_ENREF_5)] |
| E | National male condom distribution, 2012 | 251,419,268 | [[5](#_ENREF_5)] |
| F | Target for male condom distribution in Johannesburg, 2020 | 81,284,728 | Calculation: Z4*D |
| G | Male condom distribution in Johannesburg, 2012 | 24,042,996 | Calculation: Z4*E |
| H | Target for male condoms per male 15-49 in Johannesburg, 2020 | 49 | Calculation: H/Z3 |
| I | Male condoms per male 15-49 in Johannesburg, 2012 | 18 | Calculation: G/Z2 |
| J | Targeted national female condom distribution, 2020 | 40,000,000 | [[5](#_ENREF_5)] |
| K | National female condom distribution, 2012 | 4,309,146 | [[5](#_ENREF_5)] |
| L | Target for female condom distribution in Johannesburg, 2020 | 3,837,072 | Calculation: Z8*J |
| M | Female condom distribution in Johannesburg, 2012 | 413,363 | Calculation: Z8*K |
| N | Target for female condoms per female 15-49 in Johannesburg, 2020 | 2.32 | Calculation: L/Z7 |
| O | Female condoms per female 15-49 in Johannesburg, 2012 | 0.30 | Calculation: M/Z6 |
| P | Estimated target condom use at last sex with casual partner, 2020 | 80% | Calculation (figure S2) |
| Q | Condom use at last sex with casual partner, 2012 | 50% | [[6](#_ENREF_6)] |
| R | Cost per male condom | 0.80 | [[4](#_ENREF_4)] |
| S | Cost per female condom | 7.65 | [[4](#_ENREF_4)] |
| T | Annual investment to attain target condom distribution | **94,381,380** | Calculation: R*F+S*L |
|  |  |  |  |
|  | **Additional data used in calculations** |  |  |
| Z1 | Men aged 15-49, South Africa - 2012 | 14,079,521 |  |
| Z2 | Men aged 15-49, Johannesburg - 2012 | 1,346,412 |  |
| Z3 | Men aged 15-49, Johannesburg - 2020 | 1,643,853 |  |
| Z4 | Proportion of men 15-49 in Johannesburg, 2012 | 9.56% |  |
| Z5 | Women aged 15-49, South Africa - 2012 | 14,423,494 |  |
| Z6 | Women aged 15-49, Johannesburg - 2012 | 1,383,600 |  |
| Z7 | Women aged 15-49, Johannesburg - 2020 | 1,654,908 |  |
| Z8 | Proportion of women 15-49 in Johannesburg, 2012 | 9.59% |  |

Table S1: Calculations to determine the cost of scaling up the VMMC and condom distribution programs in Johannesburg

Our assumption regarding the rate of condom use at last sex with casual partner that would prevail if the target for condom distribution were met is based on the Figure S2. To construct this, we assume that in 30% of casual partnerships between males and females aged 15-49, condoms would be even if no condoms were distributed. Given that this group has an average of 20 casual acts per year, this means that condoms are used in 6 acts. We then incorporate a gradual scale-up period, on the assumption that the programmatically distributed condoms will initially not result in large increases in condom use (since people will initially replace private expenditure with publically-provided commodities). Scale-up then occurs more rapidly, with each additional condom distributed per-person assumed to cover one additional act. Finally, we assume that there will be certain proportion (20%) of acts that will never use condoms.


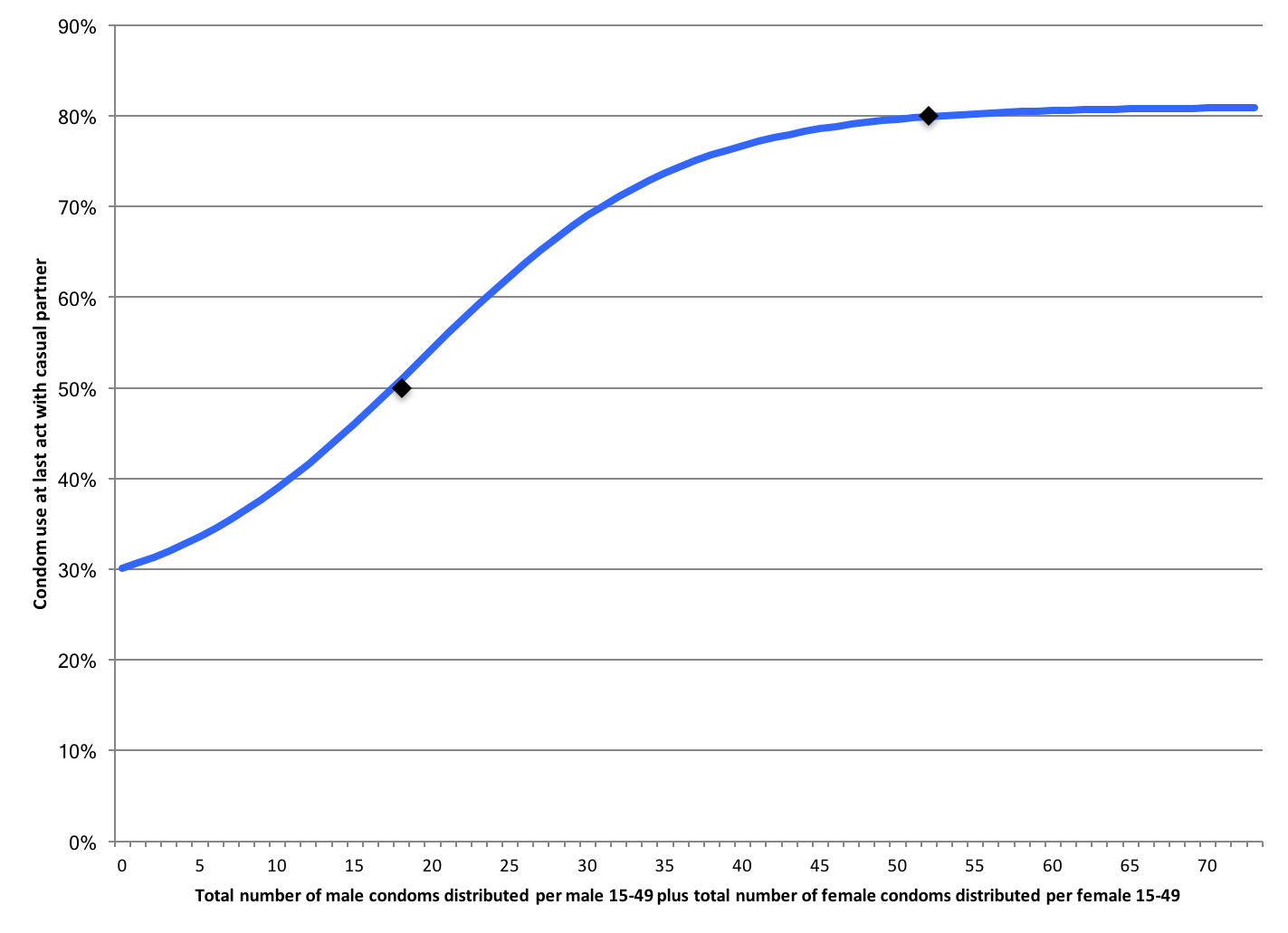


Figure S2. Assumed relationship between condom distribution and condom use at last sex with casual partner

# 4. References

1. The Optima Consortium for Decision Science, *Volume VI: Parameter Data Sources*, in *Optima HIV User Guide*. 2017.

2. Kerr, C.C., et al., *Optima: A Model for HIV Epidemic Analysis, Program Prioritization, and Resource Optimization.* J Acquir Immune Defic Syndr, 2015. **69**(3): p. 365-76.

3. SANAC, *National Strategic Plan on HIV, STIs and TB 2017-2022*. 2017.

4. Department of Health, S.A., and South African National AIDS Council, *South African HIV and TB Investment Case - Summary Report Phase 1.* 2016.

5. SANAC, *South Africa Global AIDS Response Progress Report*. 2015.

6. Shisana O, R.T., Simbayi LC, Zuma K, Jooste S, Zungu N, Labadarios D, and O.D.e. al., *South African National HIV Prevalence, Incidence and Behaviour Survey, 2012*. 2014, HSRC Press: Cape Town.
